# Toward Product Safety and Circularity: Understanding the Information Structure of Global Databases on Chemicals in Products and Articles

**DOI:** 10.1021/acs.est.4c07992

**Published:** 2025-01-26

**Authors:** Chijioke Olisah, Lisa Melymuk, Robin Vestergren, Karin Rumar, Tonie Wickman, Nina Melander, Petteri Talasniemi, Sicco Brandsma, Urban Boije af Gennäs, Martin Scheringer

**Affiliations:** † RECETOX, Faculty of Science, Masaryk University, Kotlářská 2, 611 37 Brno, Czechia; ‡ Swedish Chemical Agency (KEMI), 172 67 Sundbyberg, Stockholm, Sweden; § Swedish Centre for Chemical Substitution, RISE Research Institutes of Sweden, 114 86 Stockholm, Sweden; ∥ Finnish Safety and Chemical Agency (Tukes), FI-00521 Helsinki, Finland; ⊥ Vrije Universiteit Amsterdam, 1081 HV Amsterdam, The Netherlands; # Institute of Biogeochemistry and Pollutant Dynamics, ETH Zürich, 8092 Zürich, Switzerland

**Keywords:** consumer products, REACH, compliance, regulations, enforcement

## Abstract

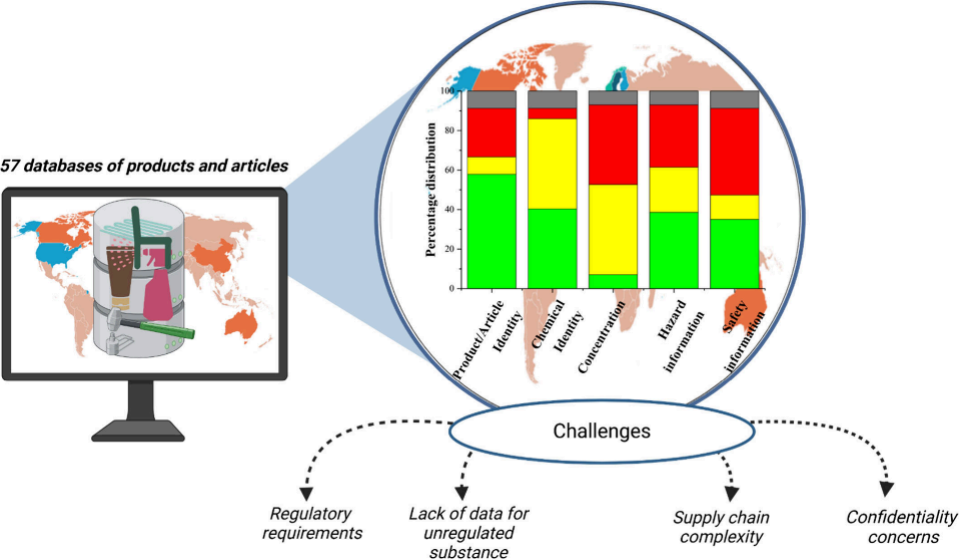

Access to information about chemicals in products and articles is critical for supporting enforcement of chemical regulations, assessing risks from chemicals, allowing informed consumer choices, and enabling product circularity. In this work, we identified and evaluated available databases (DBs) on chemicals in products and articles from the literature using a defined protocol and from European national market surveillance authorities, nongovernmental agencies, and industrial sector groups using questionnaires. This is the first comprehensive review of DBs that provide information about chemicals in products and articles. A majority of these DBs are heterogeneous in terms of scope, ontologies, and data structures. Among the 57 identified DBs, 49 identified specific substances and only 30 reported their concentration in their products. In addition, 35 DBs included hazard information and 27 DBs provided safety information about products or chemicals. The analysis highlights the lack of comprehensive or accessible data on chemicals in products and articles for most categories of products/articles and jurisdictions. The limitations of existing DBs were attributed to scattered regulatory information requirements, a lack of data for unregulated substances, the complexity of supply chain communication, and confidentiality issues. In response to these challenges, we identified opportunities for improving existing information transfer structures and exploring alternative data sources to promote product and article safety and circularity.

## Abstract 2

Unavailability, heterogeneity, and inconsistency of chemical data in product and article databases hinder risk assessment, product compliance, safety, and circularity.

## Introduction

The production of chemicals has increased exponentially in recent times. Between 2000 and 2017, global chemical production almost doubled, reaching about 2.3 billion tonnes, with rapid growth predominantly seen in China and India and this is expected to double again by 2030.^1,2^ It is estimated that over 350,000 chemicals and chemical mixtures have been registered for use globally.^3^ The Global Chemicals Outlook II report of 2019 estimated that 40,000 to 60,000 industrial chemicals are used in commerce globally.^1^ In the European Union (EU) alone, 22 459 chemical substances are registered for use in the EU market under REACH (Registration, Evaluation, Authorisation and Restriction of Chemicals).^4^ In addition to these, there are other categories of chemicals, which are not subject to registration under REACH, e.g. polymers and active substances in pesticides, biocides and pharmaceuticals. Many of the REACH-registered chemicals are used to formulate products and articles which are subsequently distributed through complex supply chains. Chemicals in chemical products and articles can be broadly categorized into intentionally added substances (IAS) and nonintentionally added substances (NIAS).^5,6^ IAS are chemicals deliberately added for specific functions, such as preservatives, colorants, or plasticizers. NIAS are chemicals present as impurities, byproducts, or contaminants. Documenting information about both IAS and NIAS is essential for regulatory compliance and ensuring the safety and efficacy of chemical products and articles.

Producers of products and articles, as well as importers, wholesalers and retailers, are responsible for the products they place on the market, and therefore, need to be aware of the chemical content of the products. Manufacturers of chemical products, cosmetics and some articles like toys also have legal duties such as labeling, providing safety information, and disclosing selected chemical contents to customers. Companies rely on chemical-related information to fulfill their obligations as well as acting proactively to stay ahead of legal requirements. Further activities depending on chemical information include innovation and product and articles development, substitution, risk assessment, scientific research, marketing, making informed choices in procurement, waste management and recycling. Nevertheless, data on chemicals in products and articles is often lacking, hindering informed decision-making.^7^ At a global scale, this lack of transparency is contrary to Targets B1 and B2 of the Global Framework on Chemicals^8^ and the overarching goal of Strategic Approach to International Chemicals Management (SAICM), which states that “information about chemicals throughout their life cycle including where appropriate, chemicals in products, is available, accessible, user friendly, adequate and appropriate to the needs of all stakeholders”.^9^ In line with these targets, the European Commission has proposed a regulation for establishing a common data platform in order to ensure that chemical data is findable and accessible.^10^ Access to the concentration of chemicals in products and articles is needed to build exposure models to support chemical risk assessment, conduct life cycle assessments, and support the transition to a circular economy.

To foster product transparency, many agencies like European Chemical Agency (ECHA), and nongovernmental organizations (NGOs), like the Environmental Working Group (EWG) have developed databases (DBs) to store data and link products and articles to their chemical composition. These DBs have become a reference asset for product and articles compliance and enforcement-related activities. In some cases, they have the ambition to go beyond existing regulations and promote substitution. Databases that contain concentrations of chemicals in products and articles can be beneficial in determining exposure levels to chemicals. Additionally, they can help pinpoint exposure triggers and develop models that can facilitate the transition to a circular economy. Assessments of information structures have been performed for chemicals using chemical inventories,^3^ and the United States Environmental Protection Agency (US EPA) has made substantial progress in developing a consumer product ingredients DB for exposure assessment.^11,12^ However, no comprehensive analysis has been completed on the information architecture of existing global DBs on consumer products and their linkage to chemicals, and this is where our current study lies.

This study identifies and critically assesses available DBs on chemical content/composition of products and articles, examining their coverage in terms of chemicals and geographical regions. We highlight gaps in the existing DB infrastructures and the challenges in the field that are limiting more comprehensive use. The definition of terms used in this paper can be found in Table S1 in the Supporting Information.

## Methods

### Selection Criteria for Consumer Product Databases

**Figure 1:**
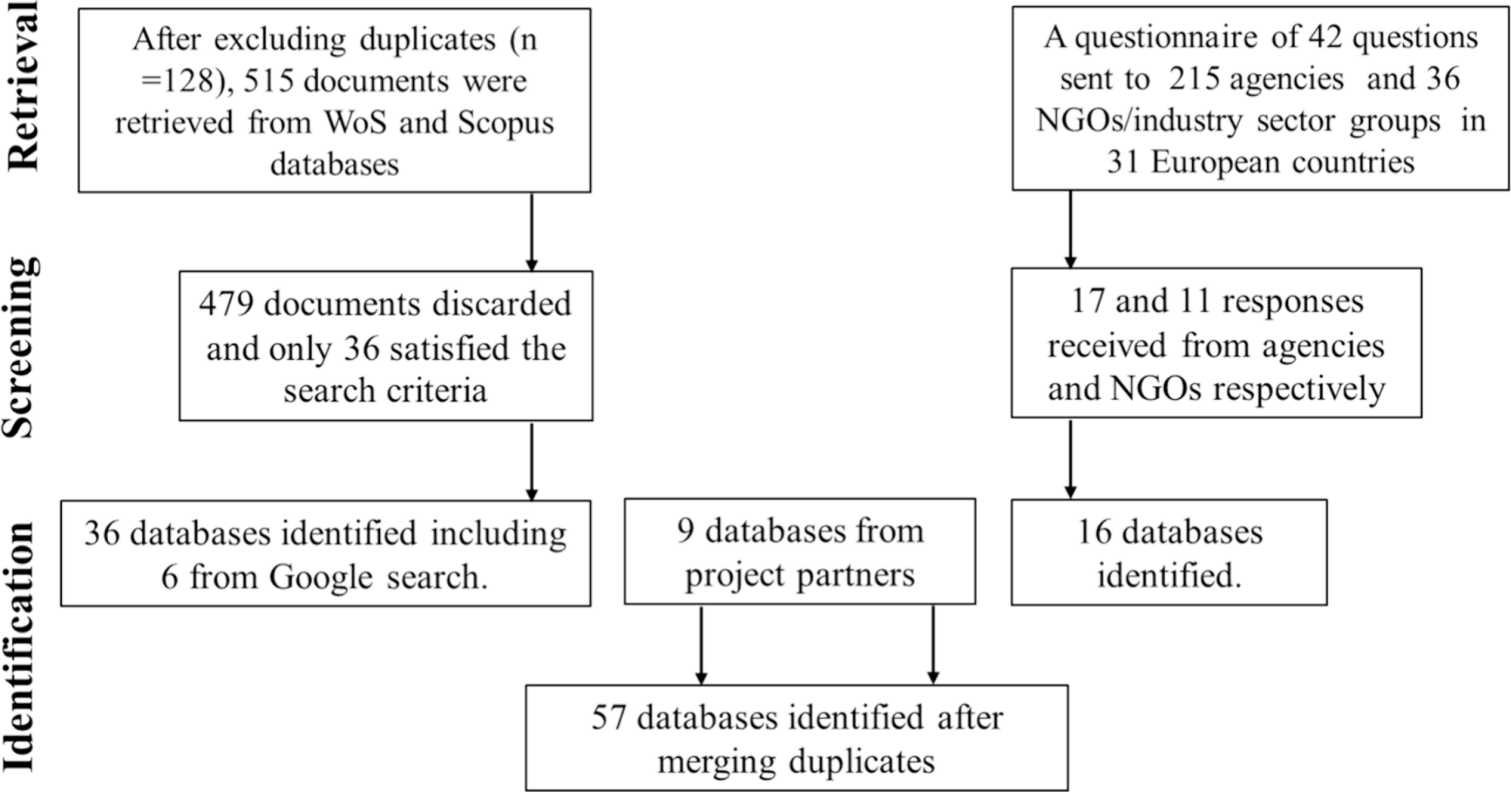
Schematic procedure of retrieval of databases.

We conducted a systematic literature search to retrieve DBs on products and articles (Figure 1). Only DBs that primarily focused on products and articles were retrieved; this included those specific to a subset of products and articles (e.g., building materials, cosmetics, toys, and biocides). Databases that focus only on chemicals without any connection to products and articles were excluded. Furthermore, we distributed a questionnaire to European national market surveillance authorities, international NGOs, and industrial sector groups to gather more information about DBs used. The study was undertaken under the European Partnership for the Assessment of Risk from Chemicals (PARC), so questionnaires were distributed predominantly to European national authorities.

### Literature Search

To identify product and article DBs in the scientific literature, we conducted a keyword search in Scopus and Web of Science (WoS). The search covered all available documents up to March 2023 on chemicals in consumer products and materials, building materials and products, certification systems, and commercial products and materials (Figure 1). We supplemented the search using Google to retrieve DBs that are not covered in scientific literature, e.g., those mentioned in gray literature and reports on product and article DBs.

### Survey Design and Data Collection

The questionnaire consisted of 42 questions divided into three sections: agency characteristics, DB availability, and enforcement activities. We only focused on responses from the first two sections, as the findings from the enforcement activities have already been reported elsewhere.^13^ The questionnaire was distributed to 215 national market surveillance authorities in 31 European countries. These selected authorities are responsible for conducting chemical management and enforcement activities in six sectors as listed by the European Commission (EC).^14^ They cover a variety of products and articles which include toys, cosmetics, biocides, chemical substances under REACH and Classification, Labeling and Packaging (CLP) regulations, other chemicals (detergents, paints, persistent organic pollutants, fluorinated greenhouse gases, ozone-depleting substances, etc.), and other consumer products under the General Product Safety Directive. Some countries have a unique agency for each sector, while others have agencies responsible for multiple sectors. The survey was also emailed to 36 international NGOs and industry sector groups with expertise related to chemical use or substances in products and articles. Information was collected between March-June 2023. The collection of information from respondents was in line with the EU General Data Protection law (Regulation (EU) 2016/679 of the European Parliament and of the Council).

## Results

### Literature Search

The literature search yielded 452 Scopus and 191 WoS documents published between 1915 to 2023. Duplicate records (n = 128) were excluded using R commands by matching the title, abstract, and keywords of the documents (Figure 1). After exclusion, a total of 515 papers were retrieved that included articles (n = 294), reviews (n = 80), short surveys (n = 78), conference papers (n = 41), and other type of documents (n = 22). These documents were reviewed, and only the 36 which had DBs mentioned in their content were screened further, yielding a total of 30 DBs with product- and article-related information. We identified six unique databases from a Google search, bringing the total to 36 databases from our literature search.

### Questionnaire Results

The overall response rate of the agency survey was 8%, representing 17 responses from 14 countries. Cyprus, Denmark, Finland, France, Greece, Ireland, Slovakia, Sweden, and the United Kingdom identified DBs. In all, ten DBs on product and articles were identified. Furthermore, we received 11 responses (response rate of 31%) from industry sector groups/NGOs. After removing duplicates, ten DBs were identified. Sixteen DBs in total were retrieved from the questionnaires from both agencies and industry sector groups/NGOs after removing duplicates, three of which were also found during the literature search.

### Overview of the Database Inventory

Combining literature searches and questionnaires, a total of 57 unique DBs were identified. Their contents were categorized with 20 descriptors covering country coverage, scope, data structure, accessibility, and number of records (Table S3). Due to inconsistent terms used to define data structures, it was challenging to categorize the DBs according to product scopes. Moreover, there is a lack of comparable ontologies, the systems of organizing data and the relationships that link data entities; recommendations have been made for such ontologies in chemistry,^15^ but such concepts have not been systematically applied regarding chemicals in products/articles. Nonetheless, to enable further analysis, DBs were classified into 14 categories. In these were 16 DBs (28%) solely classified as containing *multiple categories of products/articles*, 11 DBs (19%) solely as *biocides*, 6 DBs (11%) solely classified as *only chemicals/substances*, and 6 DBs (11%) classified as *cosmetics/personal and home care products*. Other categories with two or more DBs include *construction and building materials* (n = 4, 7%), *nanomaterials* (n = 4, 7%), and *food/food contact materials* (n = 2, 4%). The definitions of these categories are given in the Supporting Information. Of the 57 DBs, 45 are web-based, five are mobile applications, and four are Microsoft Excel spreadsheets. Most of the contents of these DBs are downloadable in either pdf or Excel format.

**Figure 2:**
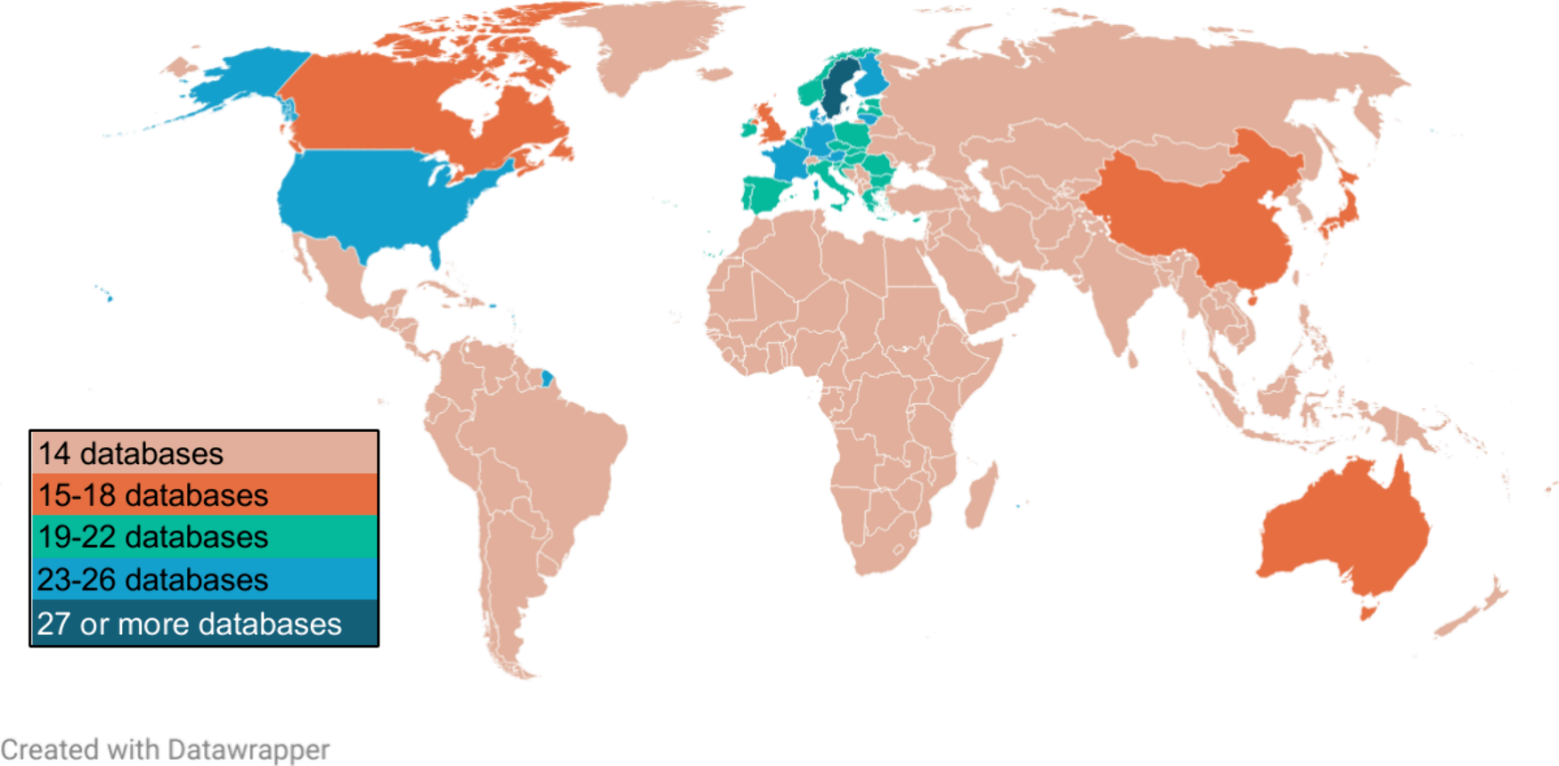
Country coverage of databases (DBs) on products and articles. Countries are color-coded according to the number of DBs. There are 14 DBs with global coverage. Detailed information about the geographic coverage is available in Table S3.

The geographical coverage of these DBs is presented in Figure 2. The highest spatial coverage was for European countries, which is likely partly influenced by the EU-focused distribution of the questionnaire, followed by the United States. Fourteen DBs have global coverage; four DBs cover Canada, one DB covers both U.S. and Europe; one DB each was identified specific to Australia, China and Japan. Ninety-one percent of DBs are publicly accessible. Five DBs have restricted access, and some of these, like the Byggvarubedömningen, contain confidential information about products that are not widely accessible. Forty-five DBs have search engines that allow users to search by product, manufacturer, keywords, or even by scanning a barcode. Some databases included chemical identifiers beyond common name (e.g., Chemical Abstracts Service Register Numbers (CAS RN), European Community Number (EC no)) (Table S3).

**Figure 3:**
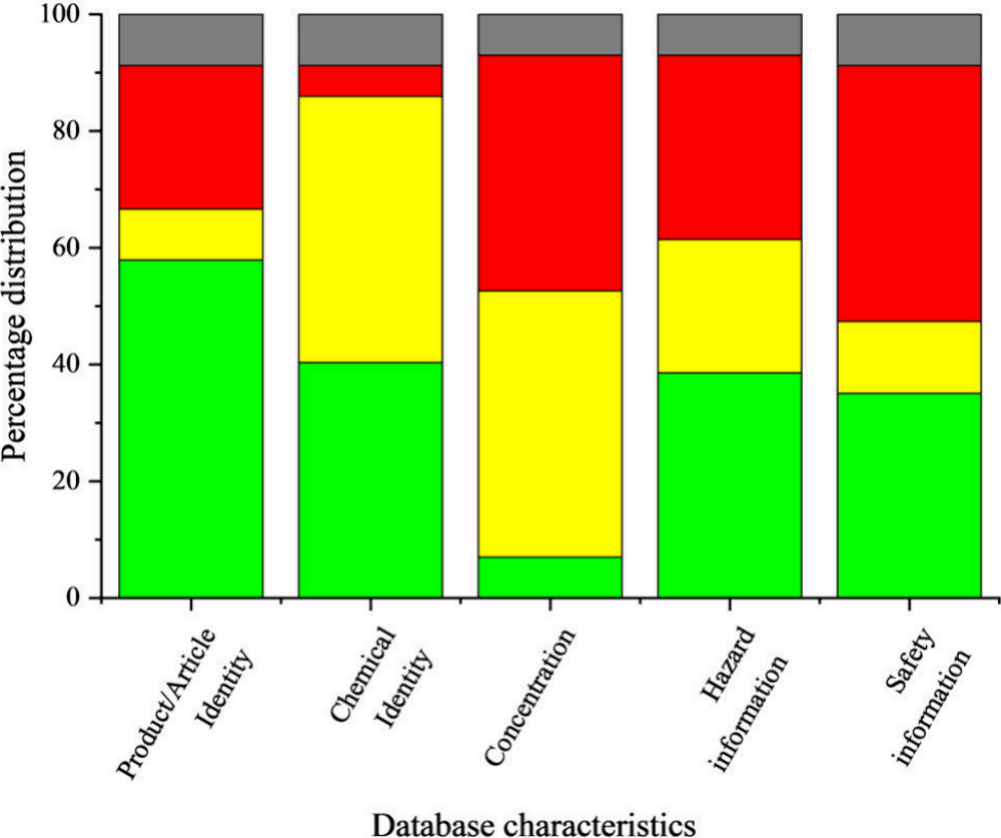
Information coverage of the 57 databases on products and articles.

Table 1 classifies the 57 DBs according to product/article scope, chemical data, and hazard and safety information, while Figure 3 summarizes the information coverage of the DBs. Of the 57 DBs, 33 (58%) identified products with brands or manufacturer information, while five (9%) specified product types without brands or manufacturer information. In terms of chemical identifiers, 49 DBs identified chemicals by name; 39 have either CAS RN or EC number, and only six indicated their InChIKey, DSSTox substance identifier, or chemical structures. Three (5%) DBs did not provide any form of chemical identification or grouping (e.g., SaferProducts and the Retailer Report Card) and 26 (46%) DBs provided nonstandard names or just a partial composition of the chemicals in products and articles (Table S3). Twenty-six (46%) DBs gave ranges or partial concentration information, while only four (7%) DBs provided full concentration profiles of the chemicals in articles and/or products. In addition, 27 (47%) of the 57 DBs had safety information about chemicals or products, and 35 (61%) contained full or partial hazard information pertaining to human and ecological impacts.

**Table 1:**
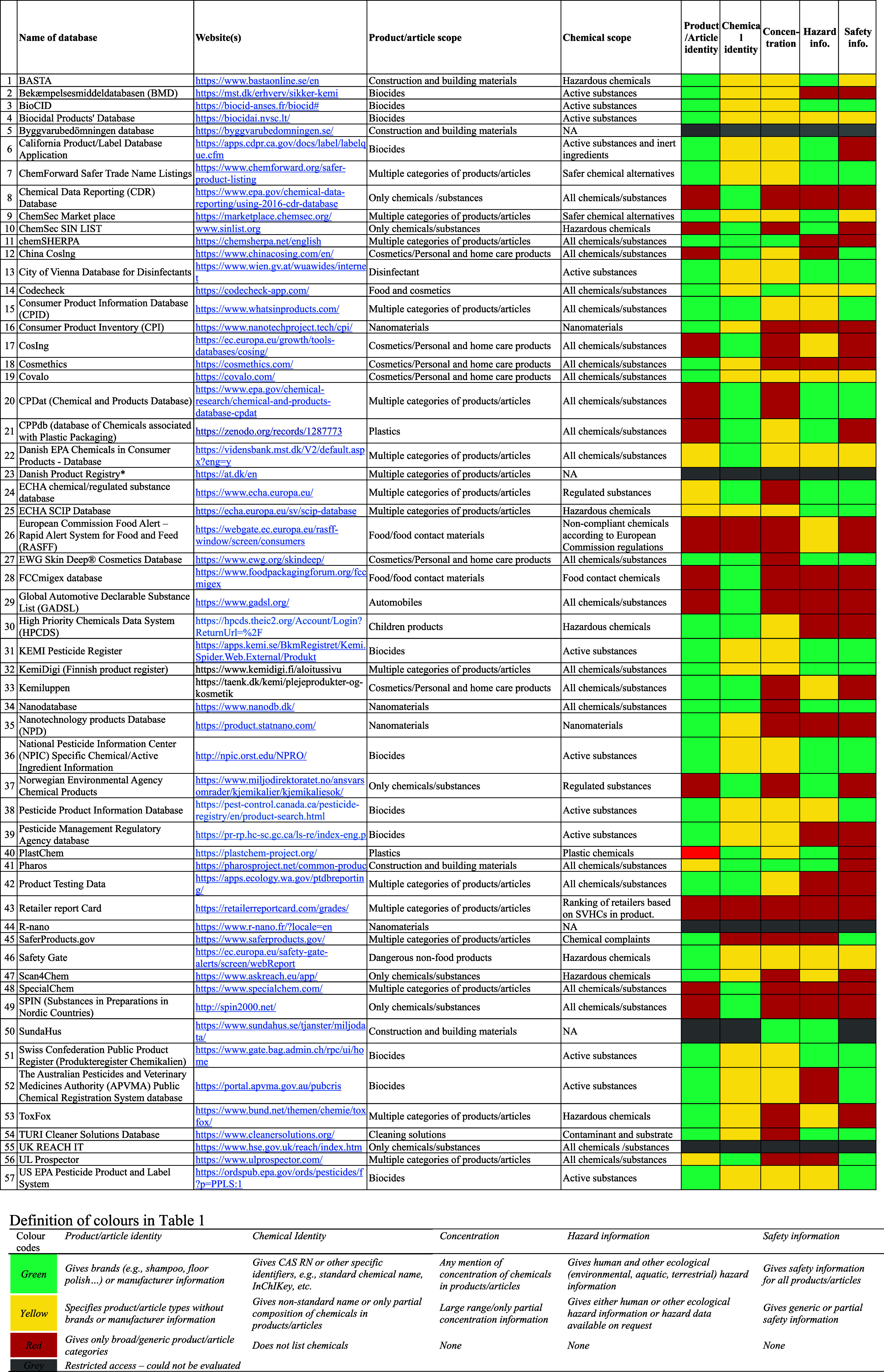
Classification of 57 DBs Using Color Codes According to Their Product Categories, Chemical Data, and Hazard and Safety Information^a^

a A detailed classification of all databases, along with definitions of categories, can be found in Table S3. NA, not accessible. Asterisks denote the publicly accessible component of the data is included under SPIN.

### Sector-Specific DBs

Some sectors have more comprehensive DB structures, in part driven by specific legislations with higher requirements for industry to provide information about product content (e.g., EU Regulation (EC) No 1223/2009 on cosmetics, EU Biocidal Products Directive 98/8/EC, the Canada Consumer Product Safety Act in Canada, Japan Industrial Standard). We discuss the structure of DBs in these sectors and other sectors like nanomaterials and building materials in more detail.

### Cosmetic/Personal and Home Care Products

To guarantee consumer safety and provide essential details to the user, cosmetics produced for the EU and many other markets must adhere to specific labeling requirements. Labeling requirements in the EU are governed by Cosmetic Products Regulation (EC) No 1223/2009 while those in the U.S. are under the Federal Food, Drug, and Cosmetic Act and the Fair Packaging and Labeling Act. Many other countries have similar regulations for cosmetics and cleaning products, e.g., the Canadian Food and Drugs Act and the Cosmetic Regulations,^16^ the Japanese Pharmaceutical and Medical Devices Law,^17^ and the Korean Quality Control and Safety Management of Industrial Products Act.^18^ The key labeling requirements of these regulations include product identity, list of ingredients, net quantity, contact details of manufacturers, and usage instructions. The nonregulatory DBs appear to be the most comprehensive. For example, the EWG Skin Deep Cosmetics DB, divided into nine product categories, contains chemical information about over 100635 products from 3656 brands. Detailed information about the products is obtained from manufacturers, online retailers, and product packaging.^19^ The Covalo DB also contains a rich data set of over 75000 beauty ingredients. Five categories are common to both the EWG Skin Deep Cosmetics and the Covalo DBs: sun, baby, fragrance, hair, face and body. The regulatory DBs, like the EU and China CosIng, are less detailed. They are more suited for cosmetic companies and regulatory purposes, and their usage requires some understanding of the EU and Chinese cosmetic regulations. They are searchable by chemical/ingredient, rather than by product, and specify if chemicals are accepted, restricted, or prohibited in cosmetics, as well as whether they can be used as UV filters, skin protectors, preservatives, or colorants.

All cosmetic DBs except EWG Skin Deep and Kemiluppen have at least a chemical name and CAS RN as a unique chemical identifier. Some chemicals in the EWG DBs are additionally linked to PubChem. All except the China CosIng and Cosmethics record hazard information about ingredients or products. EWG Skin Deep extracts the product’s ingredient list and links the ingredients with a chemical function and a chemical-specific hazard score. It uses the EWG Skin Deep scoring system to assess the health effects of products. The hazard score is generated by comparing product ingredients and Web sites to the information in toxicity and regulatory DBs.^19^

### Biocides

Most jurisdictions have established guidelines and regulations on the usage and registration of biocidal products. For example, in the EU, the Biocidal Products Regulations (BPR, Regulation (EU) 528/2012) harmonizes the rules on the supply and use of biocidal products and should ensure a high level of protection for human health, animals, and the environment. In Canada, biocides are authorized under the Food and Drug Regulations or the Pest Control Product Act but will be regulated under the Biocide Regulations as from May 31, 2025.^20^ Others include the Australian Pesticides and Veterinary Medicines Authority (APVMA) and the Federal Insecticide, Fungicide, and Rodenticide Act administered by the US EPA. Eleven DBs identified were unique to biocides. These DBs provide clear information about labeling and product classification (e.g., rodenticides, insecticides). A significant drawback of the DBs is that they record only active ingredients, and their coverage is limited to specific regions. Biocide substances in all DBs are accessible by chemical name or CAS RN. The Pesticide Management Regulatory Agency DB of Canada archived the highest number of products with >7000 records. This is followed by APVMA DB with >6500 pesticide products, and the KEMI pesticide register with >3000 products. All DBs with product information recorded the concentration of active substances. On the other hand, not all entries had complete information. For instance, in the APVMA DB, critical details such as “States of use” and “Host/pest” of some products are missing. The product type categorization was not uniform; while some products were categorized as pesticides (fungicides or herbicides), others were categorized with terms such as “active constituent” or “central nervous systems”. These inconsistencies could hinder usability of the DB. Eight of the DBs have information about product hazard assessment. The hazard information in BioCID is mainly related to allergies, skin, and eye irritation, while the Biocidal Product DB focuses on aquatic toxicity. Hazard codes are used to describe the health effects of products in the KEMI pesticide register and the California Product DB.

### Construction and Building Materials

Four DBs are specific to construction and materials. Among them, BASTA, Byggvarubedömningen, and SundaHus are Swedish DBs, with the latter two having restricted access, while Pharos primarily has U.S. coverage with limited access. Due to these restrictions, it was impossible to fully assess their content. SundaHus has the highest number of records, with >257,515 articles and 51,722 products, compared to BASTA with >180,000 articles, and Pharos with 221 products. Pharos has the highest number of chemical entries, with >178,833 chemicals, followed by SundaHus with >11,358 chemicals. Pharos has filtering functionalities that allow users to search and refine queries based on products and classification, and the chemical concentrations are linked to products. Additionally, substances, polymers, and other materials are structurally catalogued against 69 hazards, 33 restricted substances, and five endangered species lists.^21^ Many types of toxicological end points of chemicals are reported in Pharos, including carcinogenicity, mutagenicity, reproductive toxicity, and endocrine activity. Pharos uses the benchmark system of the GreenScreen for Safer Chemicals for hazard assessment.

### Nanomaterials

Advancements in the field of nanoscience are responsible for the increasing number of nanoproducts available.^22,23^ Four DBs focus exclusively on nanomaterials, and three of these are freely accessible. These DBs are targeted to stakeholders interested in incorporating nanotechnology into products, and developing strategies for the safe use of nanoproducts. The Nanotechnology Products Database (NPD), Nanodatabase and Consumer Product Inventory (CPI) have global coverage. The Nanodatabase is updated weekly,^24^ while the CPI is updated every 18 months. The NPD is the largest online nanoproduct DB with information about >10860 products from 3674 companies in 68 countries, followed by the Nanodatabase with >5000 products and the CPI with >1600 products. Entries in the NPD are grouped into 15 categories, with the *Electronic* category having the largest list of products (n = 1957), followed by *Medicine* and *Construction*. Seven product categories are common to the CPI and Nanodatabase: appliances, automotive, electronics and computers, food and beverages, goods for children, health and fitness, and home and gardens. Furthermore, the hazard profile in the Nanodatabase is in accordance with the NanoRiskCat where colors are used to indicate the effect of the nanomaterial on humans and the environment, but relies on data supplied by the manufacturer.^25^ Performing a comprehensive exposure assessment is almost impossible due to the unavailability of the concentration of nanomaterials.

A key limitation of all the nanomaterial DBs is that the application of nanomaterials or nanotechnology for product formulation is based on manufacturer claims. The term “nano” has gained much attention over the last 10 years, and some manufacturers might exaggerate the presence of nanoparticles in their products in order to increase sales.^24^ The EU has made a significant stride in this by establishing the EU Observatory for Nanomaterials (EUON) where one can search for nanomaterials in the EU market (https://euon.echa.europa.eu/search-for-nanomaterials). Substantial research has been conducted to identify and quantify nanomaterials in products,^26,27^ however, these are mostly explorative studies, limited to specific products (e.g., sunscreen) which often require sophisticated analytical tools for characterization. The lack of specialized regulations has further hampered the progress in managing the risks of nanomaterials by stakeholders. In the EU, nanomaterials are regulated under the REACH and CLP regulations, where some nanomaterials are classified by CLP as hazardous substances. They are also regulated as hazardous substances and pesticides in the U.S. EPA’s Toxic Control Act and the Federal Insecticide, Fungicide, and Rodenticide Act.

## Discussion

### Data Structure and Completeness

Together, the DBs contain relevant information that could be valuable to policymakers, manufacturers, risk assessors, enforcement authorities and researchers but the heterogeneity of coverage, structure, ontologies, and data quality limits the ability to integrate information from the different DBs. The DBs are scattered over different sources in incompatible formats with diverse searchable algorithms. Most DBs lack the organized structure to guide data users, and potential data sources used to populate specific DBs may be unknown or under-used. Many are structured and populated with varying data quality and quantity, making them difficult to use. Most DBs cover chemical products and only ten DBs (including the ECHA’s **SCIP** DBs) are on articles. It was difficult to ascertain to what extent the DBs cover chemicals in products and/or articles on the global market; DBs that indicate global coverage largely cover specific product categories in developed countries. The 14 DBs with global coverage either lack sufficient product information or are designed for specific product categories. For instance, the Nanotechnology products DB is only designed for nanoproducts used in industrial applications, while the TURI Cleaner Solution DB only focuses on chemical information about solvent cleaning. The search terms used to retrieve information in most DBs are often different. For example, the BASTA DB is searchable by article name, article number or name of company, while the Covalo DB is searchable by ingredients, claims or functions. Some require that users select from a drop-down menu to query DB data (e.g., Product Testing Data and City of Vienna DB for Disinfectants). The functionality is often specifically designed for the target audience of the DB. For example, the ChemSec SIN LIST was developed to help producers identify hazardous substances in product categories associated with their use, but not to identify specific products; Scan4Chem is targeted to consumers wanting information about the presence of SVHCs in products/articles, and does not provide information about other chemicals. Data needs often impel other DB users (e.g., researchers, enforcement agencies) to extend the use of DBs beyond their original scope and functions, despite structural limitations. Even chemical naming is a limitation in many DBs. Some DBs lacked sufficient information to identify chemicals or did not follow established chemical ontologies e.g., RASFF and Retailer report Card. More so, there are inconsistencies related to chemical spellings and nomenclature in DBs of different countries. Very few DBs include unique chemical identifiers (e.g., CAS RN, InChIKey), and the majority include only a general chemical name, in some cases only in the DB language.

Fourteen (25%) of the DBs assessed have no clear relationships between chemical and product entities. These DBs only provide information about individual chemicals or very generic product categories, and they do not link chemicals to specific products (e.g., SPIN, CosIng etc.). Those with clear linkages were designed for specific applications. For instance, the SpecialChem inventory, a large industry DB for chemical products (>350000), and the UL Prospector, another industry-oriented DB, provide information about chemical products and materials targeted toward product formulation. The ChemSec Marketplace DB only focuses on providing information about safer alternatives to materials containing hazardous chemicals, whereas the ChemForward List catalogues chemicals that are considered safe above 100 mg/kg in terms of human and environmental impacts. While these DBs may be very effective at fulfilling these specific purposes, their functionality in providing a broader understanding of the presence of chemicals in specific products is limited.

Twelve of the 16 DBs covering multiple categories of products/articles are specific to a region or country. For example, the Consumer Product Information Database (CPID) focuses on consumer products in the U.S. and Canadian markets, while the Finnish Product Register (KemiDigi), and the Danish EPA DBs focus on their national markets. The CPID appears to be the most structured of all DBs in terms of data scope and ontologies, although it only applies to household products (Table 1). This DB contains a wide variety of consumer products and links over 25000 brands and indexes the product composition and safety information extracted from ingredient lists and product safety data sheets (SDS).^28^ Products are arranged into ten categories and CAS RN and concentrations for each chemical are listed in the product composition. Additionally, the product’s brand names, and manufacturers, are provided. In terms of data scope and ontologies, this DB provides a suitable structure that could be applicable to a wider range of data users. Many DBs vary in the frequency of updates; e.g., records are added to the EU Safety Gate data weekly, however many DBs, particularly those maintained by NGOs or industry organizations, do not provide clear information about frequency of updates or whether outdated records are removed from DBs. Product formulations change over time, in response to chemical regulations as well as other aspects of product development, and the extent to which this is captured in DBs is unclear.

The ECHA chemicals/regulated substances DB, which some agencies in our survey referenced for managing products and articles, has little or no information that links chemicals to products or articles. It is developed to contain information about substances registered under REACH and substances manufactured in or imported to the EU at more than one tonne per year. This DB has been criticized for having inconsistent information about chemical identities,^29^ as individual companies provide information in the format and “shape” that these companies offer. As a result, data are still owned by the companies and the companies are responsible for their correctness and completeness. Consequently, it is unclear to what extent the data in the ECHA DB can be considered comprehensive or reliable.^30,31^ However, we note that in early 2024 ECHA launched a new chemicals DB – ECHA CHEM (https://chem.echa.europa.eu/) and chemical information is being transitioned to the new DB. The SCIP DB, on the other hand provides information about “Substances of Concern In Articles or in complex objects (Product)”. Companies supply information about articles/complex objects that contain substances of very high concern (SVHCs) (in the Candidate List) above the 0.1% w/w to ECHA to populate the SCIP DB. However, there is substantial variability in the data structure that hinders the functionalities of the DB.^32^ For instance, the information in the “Article name” column is not harmonized. The names of articles are identified in different forms, such as part numbers, alphanumeric identifiers, product descriptors, or even generic phrases (e.g., *Body in white*). Article categories are classified based on sections set out in the Council Regulation (EC) No 2658/87, and described with ambiguous nomenclatures. For instance, Section V is *Mineral Products* and Section VI is *Products of chemical or allied industries*. Also, subsectors are represented by 11-digit numbers in the “Article Category” column. As a result, users may face problems in identifying product categories. The objective of the SCIP DB as a “Dissemination Platform,″ is challenged by the complexity of its data algorithm’s user interface.

### Challenges That Limit Data Availability

A clear outcome of this analysis is the heterogeneity and inconsistency in the current landscape of DBs covering chemicals in products and articles. A robust information structure in line with the FAIR (findability, accessibility, interoperability and reusability) data principles is required to create a system that will make data available, thereby fostering product safety and circularity. In this section, we highlight four main challenges that hinder data availability in product and article DBs.

### Requirements of Regulations

The primary driver of much of the data transfer and storage related to chemicals in products and articles are regulatory requirements. Within the EU, several regulations address the content of products. For example, the composition of products like cosmetics is well-known because specific EU legislation requires the disclosure of ingredients (not mass fraction), except for impurities in raw materials used and materials used in strictly necessary quantities like solvent.^33^ In the case of cosmetics, the ingredients must be listed on the packaging in descending order by weight. Those that are present in amount less than 1% can be listed in any order at the end of the ingredients list. Regulatory requirements are generally effective in requiring data transfer on chemicals in products where necessary, however the specificity of many regulations and their geographic coverage significantly contribute to the heterogeneity of the data structures and availability of data on chemicals in products and articles. Currently, for mixtures and chemical products, information requirements under REACH are limited to SVHCs present at more than 0.1%, and disclosure requirements are limited to the name of the substance and the SDS which should contain the concentration of the hazardous substances in the mixture.^34^ Under REACH, consumers have the right to know about the presence of SVHCs in an article, its subassemblies or its packaging if it exceeds 0.1% by weight. This information should be provided free of charge within 45 days of the request but, consumers rarely use this process due to lack of awareness or the cumbersome nature of the data request process.^35,36^ Non-SVHC chemicals in articles are challenging to evaluate because there is no requirement on the composition for non-SVHC chemicals and information about their content is lacking.

To address regulatory gaps, regulation that includes a harmonized approach to disclosing information about chemicals in products and articles is necessary. This may partly be improved by regulatory actions that are already in progress globally, for example, the Global Minimum Transparency System/Standard (GMTS) is a tool that allows companies to share chemical information in materials and products throughout their life cycle in a harmonized manner.^37^ Similarly, the United Nations Environment Programme Chemicals in Product Program which aims to reduces risks of hazardous chemicals by facilitating exchange of information about chemicals in products within the supply chain.^38^ Another is the European Ecodesign for Sustainable Products Regulation (ESPR) which in principle will increase the requirements for information for substances of concern in many product categories via the digital product passport (DPP). The proposed passport will provide product information to stakeholders, thereby enhancing transparency and accountability throughout the supply chain. However, the scope of DPPs is limited to substances of concern which are defined based on hazard properties (partly overlapping with SVHC criteria) or present difficulties for recycling operators. The content of the DPP is not clear yet as negotiations are ongoing to improve its usability.^39,40^ The passport would allocate a unique ID number to each product, connecting it with the factory, supplier, and batches. Such innovation shows potential in addressing the difficulty of tracing product chemicals back to specific batches or production facilities.

### Lack of Data for Unregulated Substances

Many regulatory DBs only archive data that support regulatory compliance and decision-making processes, e.g., SVHCs or active ingredients. However, there is also a need to provide information beyond the regulatory requirements e.g. to support risk assessors and facilitate an easy transition to a circular economy. Providing information beyond just the SVHCs offers additional benefits by encouraging companies to adopt a proactive approach that goes beyond current regulatory requirements. This may include substituting or phasing out certain substances or exploring alternative materials before a substance is restricted. Such an approach also supports innovation and product development by helping companies avoid using potential SVHCs right from the product or article design stage. Furthermore, it enables downstream users, wholesalers, retailers, and consumers to make informed choices. Most of the DBs in our review did not report the actual concentrations of all chemicals in products and articles. Some DBs contain quantitative information like active ingredient(s), but the remainder of the composition is not documented, e.g., those covering biocides, UV blockers in cosmetics DBs. Most products contain more compounds beyond ingredients that must be disclosed to fulfill regulations; some DBs address this by drawing information from ingredient lists and/or SDS (e.g., EWG Cosmetics DB, CPID). However, these data sources may also lack content; more chemicals may be present in products than listed on the SDS or product labels. For example, a study found an average of 133 volatile organic compounds (VOC) in 25 products but only one of these substances are listed in the product label and two on the SDS.^41^ This lack of data may be due to the requirements for SDS/ingredient lists not being comprehensive and/or because many chemicals are unknown to the manufacturers (e.g., NIAS, impurities, reaction byproducts etc.). DB developers should seek to liaise with manufacturers and retailers to provide additional quantitative product information beyond those in the SDS, as is done by the BASTA DB system.

Information about unregulated substances that appear in the product life cycle in the form of additives, impurities, and degradation or reaction products is lacking. This gap in data prevents a comprehensive understanding of not only the effects of individual chemicals but also the cumulative risks posed by chemical mixtures, which can hinder the circularity of consumer products.^42^ Circularity can also be hindered by issues related to chemical ingredients, as some substances may be regulated or identified as hazardous with time. While the number of unregulated chemicals is unknown, the European Environmental Bureau estimated that between 5000 and 7000 chemicals will be restricted by 2030.^43,44^ Yet the EU estimates that 70,000 chemicals have poor characterization of their hazards and exposure,^45^ and one factor in the poor exposure characterization is the lack of any legal requirements mandating safety information disclosure.^44^ In the EU, this may be due to a lack of disclosure of substances in articles manufactured outside the EU or low volume substances (<10 tonnes), which are not necessarily required to have a complete risk assessment under REACH. Additionally, enforcement activities concentrate on substances that are restricted or have information requirements by performing target analysis quantifying known hazardous chemicals like brominated flame retardants and phthalate esters, leading to limited data availability for unregulated chemicals in both DBs and scientific literature. Routine product and article checks are typically done on the final product, and this may not be enough to understand the product’s composition. Also these checks target specific products and a relatively small selection of regulated chemicals,^46−50^ while nontarget and suspect screening approaches, though effective, are costly and not regularly implemented in product screening practices.^51,52^ The unavailability of high-resolution instruments and lack of training for nontarget analysis are significant challenges in implementing these workflows.

The following strategies can increase the data availability for unregulated chemicals. (i) implementing additional monitoring programs such as nontarget and suspect analysis to screen for unregulated chemicals at every stage of product formulation, not just the final product; (ii) using AI text mining techniques to retrieve unregulated chemical data from technical reports, patents, and scientific studies; and (iii) developing policies to incentivize agencies, manufacturers, and other actors to incorporate data on unregulated chemicals into existing product and article DBs.

### Complexity of the Supply Chain

Due to the complexity of the supply chain, much of the chemical data associated with the final products and articles may not be accessible to populate the DBs.^9^ This makes it challenging for manufacturers to track the composition of products from the production process to the end users. Also, information about products and articles is rarely tracked consistently unless required by sector-specific regulations, and many manufacturers are unwilling to release chemical-related data due to confidentiality concerns. During production, packaging, and distribution, chemicals can be incorporated into products and articles, and data is gathered and passed down the supply chain.^53^ However, on many occasions, there is a break in information transfer due to the complexity of the supply chain requirements and standards and lack of legal responsibility for the final products.^54^ Many industries have developed their own systems (e.g., SciveraLENS and International Material Data System for automobiles) to track chemicals along the supply chain, but such data are inaccessible outside of the industry. Addressing the complexities of supply chains has become increasingly important, particularly in the context of shifting toward green and digital economies. Since manufacturers or companies are the primary actors in generating data on products and articles, it makes sense to capitalize on their efforts to increase the amount of data on products and articles in the existing DBs. The EU DPP, currently under development, holds promise as a valuable tool for addressing complexities within product chains, particularly concerning obtaining information about chemicals in products and articles. Canada has launched consultations on mandatory labeling for chemicals in consumer products, such as cosmetics and cleaning products, to improve supply chain transparency and provide consumers with better information about the chemicals in the products they use.^55^ This initiative is part of a broader strategy to enhance the availability of information about chemicals throughout the product life cycle, supporting informed decision-making and safer alternatives. Harnessing new digital technologies like simulation techniques, AI and machine learning algorithms allows for a deeper understanding of supply chains by aiding in data integration, predictive analytics, and process automation.^56−58^ This will further provide insights that can enhance product transparency and minimize the presence of harmful chemicals in the market. The use of such digitization technologies has benefitted the automotive industry by increasing productivity, reducing inventory and improving forecasting accuracy by 80%.^59^ Additionally, there is a need to develop a system that can facilitate the easy transfer of chemical information along the supply chain, similar to the International Material Data System used in the automotive industry, where all suppliers are required to report the chemical content of their materials and components.^53^

### Confidentiality Concerns

One final factor in the lack of comprehensive data in DBs may stem from confidentiality concerns raised by manufacturers. For instance, under the REACH regulations, details such as complete mixture compositions, proprietary formulations, manufacturing processes, and trade secrets may be classified as Confidential Business Information (CBI). Approximately 18% of the total annual production values reported by EU member states in the European Union Production Statistics from 1995 to 2016 were marked as confidential.^36^ In some cases, chemical names are replaced by generic names or numbers in cases where products are covered under CBI.^60^ The ability to claim CBI status contributes to limited public access to data. Moreover, while some SDS of chemicals are accessible, some are not available to the public due to CBI claims. For instance, out of 128,700 substances identified to have safety data in chemical inventories, 43,500 have undisclosed identities, due to confidentiality claims or lack of public release.^61^ This may lead to a misconception among the general public that all chemicals in the market or used in product formulations have undergone comprehensive safety assessments by regulatory authorities. One way to ensure transparency of data while addressing confidentiality concerns may be to report chemical product categories as aggregates by grouping similar chemical products under broader categories instead of providing specific details for each product.^36^ For example, instead of reporting each individual shampoo separately, an aggregation strategy would group shampoos together and characterize average compositions or prevalence of substances of concern within a product category. An example of using the aggregation approach is the Exposure Index developed by KEMI where confidential data has been transformed to exposure indices for different target groups.^62,63^ This approach helps to simplify data transmission while still providing useful information, especially when there are confidentiality issues or when detailed information is not necessary, such as for exposure modeling and risk assessment

### Limitations

Some limitations may have hindered our retrieval of DBs. The search string inputted on WoS, Scopus and Google may not have yielded all possible studies on DBs; thus, some studies may have been left out, and the DBs presented here only reflect the query of the search. Notably, our search was conducted only in English, which may limit results from outside of English-speaking regions. Because of regulatory requirements, many countries have internal non-English databases that may not be identified by our search strategies. This is expected to largely impact product groups with stricter information requirements, e.g., biocides or cosmetics. The second limitation is the low number of responses on DB availability, and the inability to identify additional DBs that are the domain of industries and individual national regulatory agencies and are not mentioned in any scientific literature. Third, potential misclassification of DBs is possible because some DBs may belong to multiple categories. We tried to be explicit in our categorization, and this may have created some bias. Fourth, due to periodic updates, the descriptors of the DBs are likely to change over time. Therefore, this paper should be read in tandem with information about each DB Web site for a better understanding. Nevertheless, results from this study can be used to understand the nature/extent and limitations of existing chemical data on products and articles. This information serves as a useful guide for proactive efforts in chemical safety and sustainability including initiatives focused on developing safer and more sustainable products and articles, supporting substitution activities, enabling informed procurement and consumer choices, and promoting a circular economy for articles and products.

### Recommended Database Attributes

No database can possibly meet all the requirements imposed by all conceivable applications. Here, we outline the essential attributes that a product or article database should have

- The harmonized function, product and article use categories published by the Organization for Economic Co-operation and Development should be used when describing product and article categories in DBs.^64^ This provides clarity, consistency, and accessibility, which ultimately enhance the utility, accuracy, and compliance of the DB. This also enables aggregation of data across product and article groups, useful for enhancing consistency in exposure analysis.
- All chemicals (including both IAS and NIAS) in products and articles should be reported with standardized chemical identifiers, such as CAS RN, IUPAC names, and InChIKeys, along with their specific average concentrations in defined units (not ranges).
- Detailed metadata should be provided such as data source, date of entry and any relevant notes.
- The user interface for DBs should be user-friendly to allow users to find information easily and efficiently, and be searchable by multiple attributes.
- The database should be developed in such a way that it allows for future expansion and integration of new data and technologies.
- Data structure and information should conform to the FAIR data principles.

## Supporting Information Available

Glossary of terms (Table S1), search methodology (Table S2), and a complete list of evaluated databases, including access links and categorization of attributes (Table S3) (XLSX)
